# Effects of Antibiotics on Weight in Obese Patients after Sleeve Gastrectomy

**DOI:** 10.1155/2022/1592786

**Published:** 2022-09-19

**Authors:** Yisen Hou, Xinzhe Zhai, Xiaotao Wang, Yi Wu, Zhigan Lv, Peng Ma, Rui Yang, Haoliang Zhao, Jianli Han

**Affiliations:** ^1^Department of Thyroid & Bariatric Metabolic Surgery, Third Hospital of Shanxi Medical University, Shanxi Bethune Hospital, Shanxi Academy of Medical Sciences, Taiyuan, 030032 Shanxi, China; ^2^Department of Anesthesia, Third Hospital of Shanxi Medical University, Shanxi Bethune Hospital, Shanxi Academy of Medical Sciences, Taiyuan, 030032 Shanxi, China

## Abstract

Obese patients can significantly reduce weight and have a positive impact on obesity-related diseases. However, the risk of infection complications in obese people is higher than that in normal people, especially the surgical site infection. This research investigates the effect of antibiotics on weight change of obese patients after laparoscopic sleeve gastrectomy (LSG). A retrospective analysis was performed on 131 morbidly obese patients or obese patients with complications who received LSG treatment in the Third Hospital of Shanxi Medical University from 2013 to 2020. Patients were separated into the antibiotic group (59 cases) and the normal group (72 cases) according to whether antibiotics were used after surgery. The differences of postoperative weight-related indexes, inflammation-related indexes, and short-term complications were compared between the two groups. At 12-month follow-up, the % excess weight loss (%EWL) in the antibiotic group was statistically abated than that in the normal group (92.99 ± 28.60, *P* < 0.01). In addition, the percentage of total weight loss (%total weight loss (%TWL)) was abated in the antibiotic group than in the normal group, but it was not significant (*P* > 0.05). White blood cell count and neutrophil count in the antibiotic group were statistically raised than those in the in normal group (*P* < 0.05), but neutrophil/lymphocyte ratio (NLR) showed no significant difference. Comparison of short-term postoperative complications between the two groups showed that the number of abdominal wall wound infection, body temperature > 38°C, and abdominal pain > 3 days in the antibiotic group were abated, but they were not statistically significant (*P* > 0.05). Short-term antibiotic exposure after sleeve gastrectomy had an adverse effect on postoperative weight loss, with no significant improvement in short-term complications.

## 1. Introduction

According to the World Health Organization (WHO), obesity affects 13% of population, which means that the body mass index (BMI) of 650 million people is more than 30 kg/m^2^ [1]. Obesity can lead to type 2 diabetes, hypertension, hyperlipidemia, high uric acid hematic disease, and other diseases. LSG is the basic weight loss surgery; obese patients can significantly reduce weight and have a positive impact on obesity-related diseases [2]. However, the risk of infection complications in obese people is higher than that in normal people, especially the surgical site infection [3]. The Guiding Principles for Clinical Application of Antibiotics [4] pointed out that type II incision infection may exist, and antibacterial drugs should be used for prevention. The main purpose of the use of postoperative antibiotics is to prevent postoperative infection, which can reduce postoperative infection and mortality. In addition, it can avoid excessive consumption of medical resources. But some scholars at home and abroad think sleeve shape after gastrectomy antimicrobial drug use will exist for weight loss [5, 6]. Therefore, this article intends to explore the laparoscopic sleeve shape in gastric resection of antimicrobial drug use effect on the patient's body weight changes and inflammatory indexes, providing reference for clinical use antimicrobial drugs.

## 2. Clinical Data

### 2.1. The General Information

Refer to the guide [7, 8] to develop inclusion and exclusion criteria. Inclusion criteria: (1) clear diagnosis, obesity BMI > 32.5 kg/m^2^; (2) BMI > 27.5 kg/m^2^, the traditional treatment of poorly controlled and conform to at least two metabolic syndrome, or there are complications; (3) there is no history of chronic mental illness, no history of psychiatric drugs abuse, and no past surgical history of gastrointestinal surgery. Exclusion criteria: (1) physical cause or other medicines cause of obesity, including increased cortisol disease, hypothyroidism, and drug obesity; (2) there is a clear operation contraindication; (3) during pregnancy and lactation; and (4) date for any reason within six months before and after the surgery patients exposed to antibiotics.

According to inclusion and exclusion standard, from 2013 to 2020, retrospectively analyzed in Shanxi Medical University Third Hospital treated obese or morbidly obese with complications and a total of 131 patients accepting LSG, male 38 cases, and 93 cases of female. All of the patients' clinical data are complete. According to the postoperative use of antibiotics or not, they were divided into the antibiotic groups (*n* = 59) and the normal group (*n* = 72). The clinical data all came from the patient's admission review and follow-up, and all obtained the informed consent of the patients and their families.

### 2.2. Surgical Method

Surgical method reference guide is in [7, 8]. After the success of the anesthesia, regular disinfection shop towels, four-hole method operation, umbilical along the placement on the puncture outfit, establish CO_2_ pneumoperitoneum, 10 cm above the umbilicus left collarbone midline, right collarbone midline and xiphoid process under the puncture outfit were inserted into ultrasonic knife pliers, no damage. After laparoscopic exploration, along the greater curvature stomach, from broken retinal tissue, ultrasonic knife close to the wall of the stomach to cardia. His horn on the left, next to the pylorus to 4 cm, place 36 Fr stomach tube through the mouth. From the pylorus to 5 cm, use linear cutting closer against gastric tube to remove the greater curvature side of the stomach tissue, nail warehouse 5 until the bottom of the stomach, the residual stomach cavity is about 100 mL tube, using agnail can absorb line continuous slurry muscularis suture cut edge. Intraoperative protection of abdominal viscera, take out to remove the stomach tissue, no such active bleeding, gauze instruments correctly counted, eliminate pneumoperitoneum, pull out the puncture outfit, using membrane suture closure in the incision.

### 2.3. Observation Index

The basic information and weight changes of the two groups of patients before and after 1, 3, 6, and 12 months were recorded, and the changes of inflammatory indexes within 7 days were recorded. Basic information includes age, gender, height, weight, BMI, blood pressure, blood glucose, and obesity complications. Patients after discharge for 1, 3, 6, and 12 months of follow-up returned to court record these patients' body weight, BMI, %, %EWL, blood pressure, and blood sugar. Among them %TWL = (baseline weight real − time weight)/(baseline weight) by 100%; %EWL = [(baseline weight − real − time weight)]/(baseline weight − target weight) by 100%; the target weight %EWL is calculated by BMI = 25 corresponding weight value. Patients have postoperative 1-, 3-, and 7-day leukocyte count, neutrophil count, lymphocyte count, and NLR.

### 2.4. Statistical Methods

We analyzed using SPSS 21.0 software. Conform to the normal distribution of continuous numerical variables using mean difference of +/- standard deviation (^−^*x* ± *s*), with comparison between the two groups using independent sample *t*-test. Count data to [cases (%)], said line is compared between groups with chi-square test. *P* < 0.05 was statistically significant.

## 3. Results

### 3.1. Patients with General Data Comparison between Two Groups

This research included 59 patients (21 males and 38 females) in the antibiotic group and 72 patients (17 males and 55 females) in the normal group. Fasting blood sugar and glycated hemoglobin were statistically raised in the antibiotic group than in the normal group. There were no statistical differences in gender, age, height, weight, preoperative BMI, systolic pressure, diastolic pressure, 2 h PG, fasting insulin, fasting C-peptide, and hospitalization duration, as shown in Table 1.

### 3.2. Comparison of Weight-Related Indexes between the Two Groups

Comparing the antibiotic group with the normal group, 1, 3, 6, and 12 months after, the weight difference has no statistical significance. Antibiotic %TWL was abated in the antibiotic group than in the normal group from 1 month to 12 months after operation, but it was not significant (*P* > 0.05). BMI was statistically raised; %EWL was statistically abated in the antibiotic group 6 months after surgery. But BMI was no difference at postoperative 1 month and 3 months. %EWL was no difference at 1 month and 6 months after operation of two groups, as shown in Table 2.

### 3.3. Comparison of Inflammatory Indexes between Two Groups

Leukocyte and neutrophil count between two groups raised statistically in postoperative day 1, with obvious statistical significance difference. In addition, white blood cell count in postoperative day 3 difference is statistically significant. Seven days after surgery, there is no difference in leukocyte and neutrophil count between two groups. Lymphocyte count has no difference between two groups after 1, 3, and 7 days of operation. NLR between the two groups is statistically significant 7 days after surgery, as shown in Table 3.

### 3.4. Comparison of Short-Term Postoperative Complications between the Two Groups

In the antibiotic group and the normal group, postoperative gastrointestinal leakage, bleeding, venous thromboembolism, and anastomotic stenosis were not present; in the antibiotic group, abdominal wound infection, temperature > 38°C, and abdominal pain > 3 days are smaller but not significant than those in the normal group, as shown in Table 4.

## 4. Discussion

This study retrospectively analyzed 131patients with morbidly obese or obese patients with complications who received LSG in the Third Hospital of Shanxi Medical University from 2013 to 2020. According to whether antibiotics were used after surgery, the patients were separated into the antibiotic group (59 cases) and the normal group (72 cases) to explore the effect of antibiotics on the weight changes of obese patients after LSG. Our data show that there is no significant difference in total body weight in obese patients at 1, 3, 6, and 12 months after short-term exposure to antibiotics. However, the %EWL in the antibiotic group is lower than that in the normal group at 1 month after surgery and last until 12 months. In addition, the BMI in the antibiotic group is significantly higher than that in the normal group at 6 months after surgery. Therefore, we think that short-term antibiotic exposure after LSG has an adverse effect on postoperative %EWL reduction. As in previous studies, it has been found that antibiotics can destroy the intestinal flora; the negative impact of weight loss and the influence has nothing to do with the food intake, sports activities, or energy consumption [9–12]. Also, in a retrospective study [5, 6], it was found that patients treated with levofloxacin/metronidazole had significantly less weight loss than patients treated with cefoxitin 6 months after surgery, and this trend continued 12 months after surgery. However, in this clinical study, only the types of antibiotics are grouped, and there is no blank control group, so we can only speculate that the types of antibiotics will have different effects on body weight. In our study, patients who were treated with cefoxitin or cefmonate after surgery were selected as the antibiotic group and patients without exposure to antibiotics as the normal group. Compared with previous studies, the effect of antibiotic on weight change is demonstrated more scientifically.

Obesity is considered to be a chronic and systemic inflammatory disease, and its plasma clinical inflammatory markers are elevated at baseline before operation. We have the same findings in our study, which is also a reason for the prophylactic use of antibiotics. Based on this, in this study's antibiotic group with all the prophylactic use of antibiotics in the day after surgery, we found that in the first postoperative day, the antibiotic and normal group white blood cells and neutrophils were significantly elevated, and the increase in the antibiotic group was more obvious (*P* < 0.05). Lymphocytes decreased obviously. And this change will improve 7 days after surgery. Normal group of white blood cells increase associated with postoperative acute trauma, and antibiotics leukocyte and neutrophil count and the normal group rise for unknown reasons. Studies have shown that [13, 14] at postoperative day 1 WBC > 12.15 × 10^3^ *μ*/L with LSG 3.34 times associated with an increased incidence of complications, postoperative day 1 NLR 10 or higher and prolonged hospitalization, total complications, and mainly the high incidence of complications. But our data do not support this prediction model. In our observation, in both the antibiotic and normal groups, the WBC were higher than this value, the antibiotic group of postoperative day 1 NLR > 10, and normal group NLR < 10. The number of short-term postoperative complications in the antibiotic group (10 case) was less than that in the normal group (17 cases). The length of hospital stay in the antibiotic group (5.61 ± 1.53) was longer than that in the normal group (5.04 ± 2.17) (*P* > 0.05). However, some studies have shown that [15] preoperative NLR is the only risk factor for postoperative complications. In our study, there was no significant difference in preoperative NLR and postoperative complications between the antibiotic group and the normal group. Our study seems to support the conclusions of this later study.

Surgical site infection (SSI) is the most common postoperative complication [11]. According to the report, laparoscopic decrease heavy hand about 4% incidence of postoperative SSI, the most common cause of SSI microbes to *Staphylococcus aureus* and coagulase negative *Staphylococcus aureus*. Therefore, beta-lactam class antibiotics are antibiotics to prevent a line [3]. Cefoxitin is one of the most commonly used; because it is against *Staphylococcus aureus*, gram-negative bacteria and anaerobic bacteria are antimicrobial spectrum. But there is literature, which points out that the most advocate preventive gastroduodenal surgery is cefazolin [12, 16, 17]. In our study, the main antibiotics were cefamandole axetil and cefoxitin. Our study also supports this conclusion. We find that there are 7 patients (5.34%) with abdominal wound infection after surgery, including 2 (1.53%) in the antibiotic group and 5 (3.82%) in the normal group. However, there is no significant difference between the two groups (*P* > 0.05). Therefore, we believe that the use of postoperative antibiotics has no significant improvement on postoperative complications in obese patients.

There are important limitations in our research. First of all, [14, 16–18] poor postoperative body weight factors include age, male, high BMI earlier onset age, obesity, high blood pressure, and diabetes. In our study, these indicators are layered, which may affect the accuracy of experimental results. Secondly, our study did not explore the mechanism of weight change. It is reported that [10, 12, 18, 19] prophylactic use of antimicrobial drugs affects postoperative body weight change mechanism which may be associated with intestinal flora changes and bile acid. We plan to detect the intestinal flora of the patients before surgery and reexamine regularly at 1, 3, 6, and 12 months after surgery to observe the changes of intestinal flora. The changes of bile acid spectrum in serum and feces will be detected at the same time. Then, we were followed up for only 1 year, while it was reported that the effect of antibiotics on intestinal flora after LSG lasted for 1 year, that is, the intestinal flora returned to preoperation 1 year after operation. We plan to expand the follow-up period to 2 years after surgery to observe the postoperative weight changes of obese patients. Finally, the sample size of our study is insufficient, which is more obvious in the number of postoperative complications.

## 5. Conclusion

To sum up, our research suggests that LSG antibiotic use of postoperative after weight loss has an adverse effect, especially for %EWL; its mechanism may be associated with intestinal flora changes and bile acid. Postoperative antibiotic use will increase white blood cell count and neutrophil count, but no obvious improvement for short-term complications.

## 6. Summary

This research adopts the single factor analysis and comparison of antibiotic use on the sleeve shape, the influence of weight obesity patients after gastrectomy. It is found that in short-term exposure to antibiotics postoperatively, obese patients' %EWL is decreased obviously, and the overall weight change was not significant. Patients with inflammatory indexes compared with no antibiotics, leukocyte count and neutrophil count increased obviously. There was no evident difference whether use of antibiotics and short-term complications. Problems of this study were less samples, not for stratified patients with preoperative body weight. In the subsequent studies with larger sample sizes, they were divided into high BMI and low BMI groups, a longer follow-up period, and more reliable conclusions. In addition, this study did not explore the mechanism of change; in the subsequent research, we can explore the antibiotic effects on intestinal flora and bile acid changes.

## Figures and Tables

**Table 1:** Patients with general data comparison between two groups.

	Antibiotic group (*n* = 59)	Normal group (*n* = 72)	*t* value	*P* value
Gender (male/female)	21 (16.03%)/38 (29.01%)	17 (12.98%)/55 (41.98%)	2.261	0.133
Age (year)	33.39 ± 10.61	33.78 ± 7.31	-1.487	0.140
Height (m)	1.68 ± 0.09	1.67 ± 0.08	0.513	0.609
Preoperative weight (kg)	116.53 ± 23.20	110.09 ± 23.88	1.555	0.122
BMI	41.22 ± 6.66	39.16 ± 6.38	1.798	0.074
Preoperative hypertension	27 (20.61%)	28 (21.37%)		
Systolic pressure (mmHg)	158.19 ± 10.14	156.93 ± 10.28	0.456	0.650
Diastolic pressure (mmHg)	97.19 ± 10.22	99.07 ± 9.48	-0.710	0.481
Preoperative diabetes	44 (33.59%)	31 (23.66%)		
Fasting blood glucose (mmol/L)	8.74 ± 3.03	7.45 ± 1.72	2.33	0.023
2 h PG (mmol/L)	15.08 ± 5.18	13.3 ± 3.79	1.976	0.052
Glycosylated hemoglobin	7.46 ± 1.47	6.63 ± 1.09	2.812	0.006
Fasting insulin (mU/L)	36.04 ± 54.02	33.61 ± 33.43	0.222	0.825
Fasting C-peptide (nmol/L)	5.16 ± 5.11	5.31 ± 2.18	-0.155	0.877
Hospitalization time (day)	5.61 ± 1.53	5.04 ± 2.17	1.694	0.093

**Table 2:** Comparison of weight-related indexes between the two groups.

Index	Group	Preoperative	1 month after operation	3 months after operation	6 months after operation	12 months after operation
Weight (kg)	Antibiotic group	116.53 ± 23.20	105.24 ± 19.73	95.24 ± 16.52	88.26 ± 15.51	81.89 ± 14.36
	Normal group	110.09 ± 23.88	99.38 ± 21.66	90.48 ± 20.43	82.92 ± 18.60	76.63 ± 16.98

BMI	Antibiotic group	41.22 ± 6.66	37.24 ± 5.53	33.73 ± 4.67	31.27 ± 4.42^∗^	28.98 ± 3.80^∗^
	Normal group	39.16 ± 6.38	35.36 ± 5.85	32.19 ± 5.58	29.52 ± 5.18^∗^	27.30 ± 4.85^∗^

%TWL	Antibiotic group		9.43 ± 2.75	17.71 ± 5.07	23.62 ± 6.52	28.95 ± 7.97
	Normal group		9.69 ± 2.62	17.81 ± 3.98	24.57 ± 5.20	30.14 ± 6.45

%EWL	Antibiotic group		25.70 ± 7.02^∗^	48.35 ± 12.91^∗^	64.86 ± 19.12^∗∗^	79.28 ± 22.95^∗∗^
	Normal group		29.91 ± 11.51^∗^	55.09 ± 18.43^∗^	75.83 ± 23.49^∗∗^	92.99 ± 28.60^∗∗^


^∗^Comparison between the antibiotic group and the normal group, *P* < 0.05; ^∗∗^comparison between the antibiotic group and the normal group, *P* < 0.01.

**Table 3:** Comparison of inflammatory indexes between two groups.

Index	Group	Preoperative	1 day after operation	3 days after operation	7 days after operation
White blood cells (10^9^/L)	Antibiotic group	8.16 ± 1.93	14.57 ± 3.98^∗∗^	10.85 ± 2.79^∗^	8.33 ± 2.02
	Normal group	7.80 ± 1.59	13.04 ± 2.03	9.97 ± 1.36	8.31 ± 1.19

Neutrophils (10^9^/L)	Antibiotic group	5.04 ± 1.62	12.24 ± 3.63^∗∗^	7.99 ± 2.22	5.56 ± 1.68
	Normal group	4.58 ± 1.26	10.73 ± 1.88	7.37 ± 1.27	5.31 ± 1.02

Lymphocyte (10^9^/L)	Antibiotic group	2.51 ± 0.72	1.28 ± 0.46	1.83 ± 0.63	2.11 ± 0.72
	Normal group	2.49 ± 0.66	1.26 ± 0.41	1.80 ± 0.47	2.29 ± 0.53

NLR	Antibiotic group	2.12 ± 0.79	10.65 ± 4.33	4.72 ± 1.67	2.94 ± 1.46^∗^
	Normal group	1.95 ± 0.82	9.42 ± 3.41	4.39 ± 1.52	2.44 ± 0.83


^∗^Comparison between the antibiotic group and the normal group, *P* < 0.05; ^∗∗^comparison between the antibiotic group and the normal group, *P* < 0.01.

**Table 4:** Comparison of short-term postoperative complications between the two groups.

Group	Gastrointestinal leakage	Hemorrhage	Venous thromboembolism	Anastomotic stenosis	Abdominal wound infection	Body temperature > 38°C	Abdominal pain > 3 days	Total number of complications
Antibiotic group	0	0	0	0	2 (1.53%)	3 (2.29%)	5 (3.82%)	10 (7.63%)
Normal group	0	0	0	0	5 (3.82%)	5 (3.82%)	7 (5.34%)	17 (12.98%)
*χ* ^2^ value					0.260	0.006	0.061	0.880
*P* value					0.610	0.940	0.805	0.348

^∗^Comparison between the antibiotic group and the normal group, *P* < 0.05; ^∗∗^comparison between the antibiotic group and the normal group, *P* < 0.01.

## Data Availability

The labeled dataset used to support the findings of this study is available from the corresponding author upon request.
